# Signaling sickness: the role of recalled sickness behavior and psychosocial factors in shaping communication style

**DOI:** 10.1093/emph/eoab017

**Published:** 2021-06-11

**Authors:** Eric C Shattuck, Jessica K Perrotte, Colton L Daniels, Xiaohe Xu, Thankam S Sunil

**Affiliations:** Institute for Health Disparities Research, University of Texas at San Antonio, One UTSA Circle, San Antonio, TX 78249, USA; Department of Public Health, University of Texas at San Antonio, One UTSA Circle, San Antonio, TX 78249, USA; Department of Psychology, Texas State University, 614 N. Guadalupe St. #253, San Marcos, TX, USA; Department of Sociology, University of Texas at San Antonio, One UTSA Circle, San Antonio, TX 78249, USA; Department of Sociology, University of Texas at San Antonio, One UTSA Circle, San Antonio, TX 78249, USA; School of Public Administration, Sichuan University, Yuzhang S. Rd., Jiuyan Bridge, Wuhou District, Chengdu, Sichuan, China; Department of Public Health, University of Tennessee, Knoxville, 1914 Andy Holt Ave, Knoxville, TN 37996, USA

**Keywords:** sickness behavior, signaling, stoicism, health communication, infectious disease

## Abstract

**Background and objectives:**

Active infection results in several outward signs in humans, including visible symptoms, changes in behavior and possible alterations in skin color and gait. A potential adaptive function of these indicators is to signal distress and elicit care from close others. We hypothesized that sickness behavior, a suite of stereotypical changes in mood and behavior, also serves to communicate health status to others. We further hypothesized that such outward signals/cues of health status would vary based on context and sociocultural norms.

**Methodology:**

We explored self-reported, recalled sickness behavior, communication style, demographics and theoretically relevant cultural factors in a large national US sample (*n* = 1259) using multinomial probit regressions.

**Results:**

In accordance with predictions, relatively few participants were willing to talk or complain about sickness to strangers. Self-reported, recalled sickness behavior was associated with some communication styles but attention received from others was more consistently associated with potential signaling. Several cultural factors, including stoicism and traditional machismo, were also associated with different sickness signaling styles.

**Conclusions and implications:**

These preliminary, self-reported data lend some tentative support to the sickness behavior signaling hypothesis, though experimental or observational support is needed. The role of cultural norms in shaping how such signals are transmitted and received also deserves further attention as they may have important implications for disease transmission.

**Lay Summary:**

Evolutionary medicine hypothesizes that signs and symptoms of infectious disease—including sickness behavior—have adaptive functions, one of which might be to reliably signal one’s health status to others. Our results suggest that evolved signals like these are likely shaped by cultural factors.

## INTRODUCTION

Signs and symptoms are one of the hallmarks of infectious disease, are critical for differential diagnosis and disease tracking, and are the target of many therapeutic treatments. A major contribution of evolutionary medicine is the recognition that signs and symptoms often benefit hosts and pathogens, rather than being the inconvenient byproducts of infection [1]. For instance, coughing and sneezing can clear the throat and nasal passages of pathogens while also aiding disease transmission.

Symptoms may also function to communicate sickness and infectivity on a group level. This is a key premise of the putative behavioral immune system, whereby salient indications of infectious disease lead to avoidance behavior, or, intriguingly, elevated immunological responses in healthy individuals [2]. For instance, Schaller *et al.* [3] found that showing pictures of visibly sick individuals (e.g. coughing and holding a tissue) was associated with increased *in vitro* interleukin-6 (IL-6) release from lipopolysaccharide (LPS) stimulated white blood cells when compared to individuals who had seen neutral or non-disease related threat images.

Sneezing, coughing, rhinorrhea, red eyes and other such symptoms are undoubtedly obvious indicators of sickness and generally occur later in the course of the illness. Experiments have shown that even the activation of the inflammatory response—absent any replicating pathogen—has observable effects on behavior and physiology within a brief timespan (i.e. 1–2 h). Following LPS administration, facial skin became lighter and less red in a sample of non-Hispanic Whites, which is consistent with other studies showing that paler, less red skin indicates poor health in individuals with white skin [4]. Experimental inflammation due to LPS has also been linked with increased sighing and deep breathing in men but not women, as well as an increase in moaning and verbal complaining at low frequencies [5]. Gait speed is also reduced during inflammation and innate immunity activation, which independent raters judged as indicating poor health [6]. Taken together, these results indicate that there is an early, multifaceted physiological and behavioral response to inflammation that can communicate health status to others through movement, skin coloration, breathing patterns and vocalizations. These responses may have public health implications if healthy individuals can attend to them and reliably alter their behavior to avoid the sick. 

In addition to these changes in physiological characteristics, sickness behavior may also communicate infection. Sickness behavior occurs early after infection and is tightly linked with inflammation through several pro-inflammatory cytokines [7]. Some of the behavioral and mood changes collectively classified as sickness behavior include increased lethargy, depressed affect, changes in diet and cognition, social withdrawal and decreased libido [8]. The leading explanation for this phenomenon is that it is an adaptive response on the part of the host to conserve energetic resources through a reprioritization of behavior, thereby ensuring that more energy can be devoted to maintaining a vigorous immune response [9]. All things being equal, this should result in increased survival and reduced convalescence time. However, the demonstrable changes in behavior can also serve to advertise health status. Whether this communicative aspect of sickness behavior fits the definition of a signal (characteristics evolved with the specific purpose of communicating information) or a cue (a byproduct of another process or activity that happens to transmit information but which has not evolved for that purpose) remains to be determined [10]. Sickness behavior does seem to fit other characteristics of a true signal, however, namely that it is produced by the signaler, elicits a response in the receiver and may result in positive fitness consequences for signaler and recipient [10]. For instance, house finches reduced the time spent with partners displaying sickness behavior (increased fluffing and decreased locomotion) than with healthy control partners [11]. While there are numerous studies linking avoidance with infection in diverse taxa, this is the only study we are aware of to find that avoidance is associated with sickness behavior, rather than chemical cues, specific alarm behaviors, or other cues [12]. While there is likely no direct fitness benefit for the signaler if sickness behavior elicits avoidance, there can be a fitness benefit to the recipient, hence kin selection or altruism may play a role in explaining this avoidance [13]. On the other hand, sickness behavior may elicit caregiving behaviors with a direct fitness benefit for a sick individual [14].

Any relationship between sickness behavior and elicitation of caregiving must strike a fine balance between the benefits of that care for the sick individual and potential costs associated with exposing a caregiver to a pathogen. Additionally, while sickness behavior is thought to improve recovery and survival, it is not without its own costs. In nonhuman animals, lost opportunities for reproduction or offspring care are likely among the most salient opportunity costs [15]. In humans, these opportunity costs could include lost wages at work and reduced child care, among others. It is also likely that sickness behavior incurs its own energetic costs through reduced food intake. Taken together, it is plausible that caregiving can help offset these costs by increasing available energy through provisioning, shortening the convalescence period even further through direct care (e.g. wound cleaning in animals or therapeutic care in humans), indirect immunological benefits of companionship, or by assisting with other duties, such as alloparenting or childcare in humans [16].

There is experimental evidence showing that inflammation is associated with stronger desires to be around supportive others through increased activity in the ventral striatum, an area of the brain strongly implicated in reward processing [17]. These results echo findings in non-human animals, where sick or inflamed individuals increase affiliation with familiar cage-mates [18]. Changes in social behavior and contact therefore likely depend on familiarity and relationship context. Among friends and family, communicating sickness could elicit care (and associated close contact), while among strangers, the same information would more likely result in avoidance. It may also be disadvantageous to express sickness behavior outside of kin and friends, especially in the context of competition. This is supported by evidence from nonhuman animals, including the expression of sickness behavior in solitary, but not group-housed, zebra finches and in dominant, but not subordinate, mice [19, 20]. While we are not aware of any studies explicitly connecting sickness behavior to caregiving, there are parallels with pain, another signal of distress [14, 21]. Expected empathic reactions and strong support networks have been linked with worse reported pain and greater pain expression [22,23].

Finally, sickness behavior and its expression are expected to be at least somewhat culturally constructed [24]. Previous research has shown that cultural norms and values that shape multiple aspects of sickness and health also affect self-reported, recalled sickness behavior [25]. These factors could similarly influence sickness signaling by affecting when, and to whom, it is appropriate to share feelings of sickness or display sickness behavior. For instance, stoicism and notions about responsible use of medical services influenced the timing of medical consultations for symptoms later determined to be lung cancer [26]. Notably, though stoicism is often equated with masculinity, both men and women in this study often filtered their cancer experience through a stoic lens. Nor is stoicism limited to adults. Using vignettes about physical symptoms, adolescents frequently responded that a fictional character with a stomachache would be more apt to hide their discomfort among peers for fear of being teased or perceived as weak [27]. Relatedly, some masculine norms that are characterized by hypermasculine ideals may favor other stoicism-like behavioral scripts, such as self-sufficiency and emotional control that may inhibit displaying symptoms [28]. On the other hand, adherence to a more feminine gender role was associated with increased physical symptom reporting in both men and women [29].

Familism, a set of values rooted in familial support and obligation, has also been associated with the adoption of a sick role [30]. It is possible that people who are oriented toward familism may feel more comfortable signaling sickness to close family and may also feel as though they will be supported by family during sickness. Individualism and collectivism similarly shape beliefs about sickness, approaches to doctor–patient interactions and health communication [31]. Finally, coping strategies may also influence sickness signaling, with distraction coping predicting stronger physical symptoms of heart failure [32]. On the other hand, active coping—the degree to which one confronts problems directly—is associated with reduced somatic symptoms and improved physical health functioning and self-rated health [33]. The sociocultural milieu clearly shapes perceptions of sickness and appropriate disclosure of symptoms, much as it influences perceptions of and interactions with the world more generally. Therefore, any signaling function of sickness behavior or other signs of infectious disease is likely filtered through these sociocultural lenses which could have downstream consequences for disease transmission if signals are covered up or ignored.

We included questions exploring the communication function of sickness behavior in a large nationwide survey addressing multiple elements of sickness behavior. Based on the hypothesis that sickness behavior serves a signaling function, we predicted that participants would be more likely to express or otherwise communicate sickness around friends and family than around strangers and that self-reported, recalled sickness behavior would be associated with communication to friends and family, as would the amount of perceived attention received by the participant when sick (i.e. operant conditioning). Given the role of cultural norms and values in shaping self-reported sickness behavior, interpretations of symptoms and disclosure of health status, we also include several theoretically relevant sociocultural factors as covariates to determine their incremental effects on sickness communication. Specifically, we examine the potential effects of stoicism, traditional machismo, gender role, individualism and collectivism, active coping and familism (Table 1). While the literature on sickness communication within different sociocultural contexts is sparse at best, we tentatively predict that stoicism, individualism, traditional machismo and active coping would be associated with decreased willingness to express sickness to others and that a more feminine gender role, collectivism and familism would be associated with increased communication.

**Table 1.** Scales and sample items used in this study

Scale Name	Sample Item	Alpha
Traditional Masculinity-Femininity Scale [34]	Traditionally, my outer appearance would be considered as [masculine/feminine]	0.97
Traditional Machismo and Caballerismo Scale [35]	A man should not cry in front of his children	0.92
Familism [36]	I cherish the time I spend with my relatives	0.92
14-item Individualism/Collectivism Scale [37]	I enjoy being unique and different from others in many ways; I usually sacrifice my self-interest for the benefit of my group	Individualism: 0.82
		Collectivism: 0.86
John Henryism Active Coping Scale [38]	When things do not go the way I want them to, that just makes me work even harder	0.89
Pathak–Wieten Stoicism Ideology Scale [39]	I believe my physical pain is best handled by just keeping quiet about it	Endurance of pain: 0.72
		Taciturnity: 0.71
SicknessQ [40]	I do not wish to do anything at all; I wish to be alone	0.91

## METHODOLOGY

Data come from an online survey distributed through Qualtrics in November of 2018. Participation was open to all non-Hispanic White, non-Hispanic Black or Hispanic US adults between the ages of 18–55 who self-identified as having been sick within the past year. All ethical approvals were obtained (IRB #19-020E), as was informed consent. Qualtrics distributed the survey and recruited and screened all participants. Further details about screening and the survey response rate can be found in our previous publication [25]. Ethnicity and gender counts were: 429 Whites, 421 non-Hispanic Blacks, 409 Hispanics, with 629 men and 630 women. Mean age was 36 years, median income category was $40 000–$49 999, and median education was ‘some college’.

Scales measuring norms and beliefs used in this analysis are described in Table 1. In all cases, a higher score indicates a stronger adherence to that norm/belief. The SicknessQ scale measures sickness behavior and has been validated during experimental inflammation studies [40]. In our survey, participants were asked to think about recent times they had been sick with minor infectious illnesses (e.g. common cold and influenza) and complete the SicknessQ based on that experience. To assess whether participants believed that they received more attention from others when sick, we used a question from the Illness Cognitions Scale [41]. Participants rated their agreement with the statement, ‘people give me more attention when I am ill’, on a 5-point scale from strongly disagree (1) to strongly agree (5). The unweighted mean value was 2.97 (std. dev. = 1.31). This attention is central to a successful signaling strategy and, as noted above, may also help reinforce the use of such signaling through operant conditioning.

Finally, to explore possible signaling behaviors during sickness, participants were asked several true/false questions related to their behavior and sensations around friends/family and strangers. These were, ‘I am more likely to feel worse physically when around (friends or family/strangers) than when I am alone’, ‘I am more likely to exaggerate the severity of my symptoms or complain about them when around (friends or family/strangers) than when I am alone’, and ‘I often talk about or mention my symptoms when around (friends or family/strangers)’. For each possible signaling ‘domain’ (increased subjective feelings of sickness, exaggeration/complaining and talking about symptoms), participants were coded as not signaling to both friends/family and strangers (‘taciturn’), signaling to only friends/family, signaling to only strangers, and signaling to both friends/family and strangers (‘gregarious’). These categories were theoretically informed by research suggesting that sickness behavior expression (i.e. expression of the signal or cue) is dependent on social contexts [19, 20].

All analyses were conducted in STATA 14 [42]. Multinomial probit regressions were used to determine how demographics, SicknessQ scores, attention scores and beliefs and values affected signaling style in each domain. Results are expressed as exponentiated coefficients which correspond to probability changes in being classified as ‘taciturn’ or as engaging in other communication behavior. A multinomial probit model is an alternative to a multinomial logit model that relaxes the independence of irrelevant alternatives (IIA) assumption inherent in the latter. This assumption holds that any choice between two alternative options in the outcome variable should be independent of the presence of a third option. To avoid the possibility of Type 1 errors in these models, significance was set at 0.01.

## RESULTS

Table 2 shows the unweighted crosstabulations for each signaling domain and style of the signal. Notably, there are very few individuals who advertise illness to strangers, with the exception of subjectively feeling worse. Additionally, the majority of people indicated that the presence of both friends/family and strangers had no effect on their behavior or sensations when sick.

**Table 2.** Cross-tab frequencies across communication domains

I often talk about or mention my symptoms when…		
	…around strangers	
…around friends/family	False	True
False	600 (48%)	64 (5%)
True	336 (27%)	258 (20%)

I am more likely to exaggerate the severity of my symptoms or complain about them when…		
	…around strangers	
…around friends/family	False	True
False	783 (62%)	75 (6%)
True	156 (12%)	244 (19%)

I am more likely to feel worse physically when…		
	…around strangers	
…around friends/family	False	True
False	641 (51%)	156 (12%)
True	153 (12%)	308 (24%)

Full results of the baseline models (adjusted for demographics but not sociocultural variables) can be found in the Supplementary Material. Relative to taciturn individuals, greater sickness behavior was associated with a slight increase in the probability of exaggerating sickness when around strangers (exp.(b) = 1.031, or 3.1% increase) and with feeling subjectively worse around friends/family (exp.(b) = 1.034, or 3.4% increase) and both friends/family and strangers together (exp.(b) = 1.040, or 4% increase). Though we did predict that sickness behavior would be associated with a greater probability of signaling to close others, its association with signaling to strangers is contrary to predictions. The attention received from others when sick was more consistently and more strongly associated with signaling in all domains (Supplementary Material).

**Figure 1.**
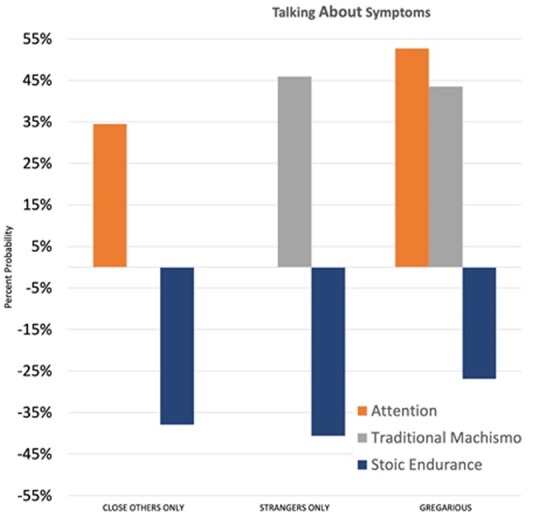
Significant predictors of communication style, talking about symptoms.

Tables 3, 4 and 5 show the results of the fully adjusted models. With regard to willingness to discuss sickness, we found that people who believed that they received more attention when sick were more likely to talk about sickness with friends and family or adopt a ‘gregarious’ communication style (Table 3, Fig. 1). There was no role for recalled sickness behavior on communication style at the 0.01 significance threshold, though it was associated with gregariousness at *P* < 0.05. Stoic endurance of pain and illness was uniformly associated with a decreased likelihood of talking about sickness with others (Table 3, Fig. 1). Additionally, traditional machismo was associated with an increased likelihood of talking about sickness with strangers (exp.(b) = 1.459 or 45.9% increase) or both strangers and friends/family (exp.(b) = 1.435 or 43.5% increase). In terms of demographics, older age was associated with a decreased likelihood of talking about sickness to strangers only (exp.(b) = 0.957, or 4.3% decrease).

**Table 3.** Results of fully adjusted model, talking about symptoms

Talking about symptoms						
	Friends/family		Strangers		Gregarious	
	Coef. (SE)	Exp (coef.)	Coef. (SE)	Exp (coef.)	Coef. (SE)	Exp (coef.)
Age	−0.014 (0.009)	0.986	−0.044 (0.013)	0.957^**^	−0.010 (0.010)	0.990
Female	0.146 (0.251)	1.157	−0.198 (0.301)	0.821	−0.702 (0.280)	0.496^*^
Non-Hispanic Black	−0.102 (0.196)	0.903	−0.295 (0.290)	0.745	−0.249 (0.230)	0.779
Hispanic	−0.134 (0.203)	0.870	−0.478 (0.299)	0.620	0.016 (0.239)	1.016
Currently married	0.174 (0.182)	1.190	0.464 (0.255)	1.590	0.434 (0.220)	1.544^*^
SicknessQ	0.023 (0.012)	1.023	0.015 (0.015)	1.015	0.030 (0.012)	1.030^*^
Sick attention	0.296 (0.074)	1.345^**^	0.067 (0.087)	1.070	0.423 (0.093)	1.527^**^
Gender role	0.004 (0.066)	1.004	0.059 (0.075)	1.061	0.107 (0.064)	1.113
Machismo	0.060 (0.074)	1.062	0.378 (0.100)	1.459^**^	0.361 (0.080)	1.435^**^
Familism	0.069 (0.110)	1.072	−0.385 (0.166)	0.681^*^	−0.181 (0.111)	0.834
Individualism	0.131 (0.071)	1.140	0.202 (0.095)	1.224^*^	0.138 (0.066)	1.148^*^
Collectivism	0.109 (0.071)	1.115	0.127 (0.101)	1.136	0.199 (0.067)	1.221^*^
John Henryism	−0.011 (0.012)	0.989	−0.006 (0.015)	0.994	−0.011 (0.013)	0.988
Stoic endurance	−0.477 (0.108)	0.620^**^	−0.520 (0.150)	0.594^**^	−0.314 (0.120)	0.730^**^
Stoic taciturnity	−0.110 (0.117)	0.896	0.239 (0.130)	1.270	−0.149 (0.116)	0.861

*Note*:

* *P* ≤ 0.05,

** *P* ≤ 0.01.

**Table 4.** Results of fully adjusted model, exaggerating/complaining about symptoms

Exaggerating/complaining about symptoms						
	Friends/family		Strangers		Gregarious	
	Coef. (SE)	Exp (coef.)	Coef. (SE)	Exp (coef.)	Coef. (SE)	Exp (coef.)
Age	−0.026 (0.010)	0.974^**^	−0.014 (0.011)	0.986	−0.027 (0.011)	0.973^**^
Female	−0.100 (0.297)	0.904	−0.362 (0.310)	0.696	−0.904 (0.257)	0.405^**^
Non-Hispanic Black	0.003 (0.235)	1.003	0.201 (0.251)	1.222	−0.622 (0.228)	0.537^**^
Hispanic	0.054 (0.235)	1.056	−0.276 (0.253)	0.759	−0.332 (0.249)	0.718
Currently married	0.450 (0.194)	1.568^*^	0.651 (0.231)	1.918^**^	0.418 (0.213)	1.519^*^
SicknessQ	0.014 (0.012)	1.014	0.036 (0.013)	1.036^**^	0.006 (0.011)	1.006
Sick attention	0.280 (0.088)	1.323^**^	0.295 (0.096)	1.342^**^	0.321 (0.096)	1.379^**^
Gender role	0.019 (0.072)	1.020	0.008 (0.086)	1.008	0.132 (0.060)	1.142^*^
Machismo	0.231 (0.089)	1.260^**^	0.319 (0.095)	1.375^**^	0.351 (0.078)	1.420^**^
Familism	0.155 (0.112)	1.167	−0.009 (0.122)	0.991	0.034 (0.124)	1.034
Individualism	−0.055 (0.094)	0.947	0.076 (0.084)	1.079	0.075 (0.070)	1.078
Collectivism	0.039 (0.087)	1.039	0.032 (0.074)	1.032	0.107 (0.070)	1.113
John Henryism	−0.031 (0.013)	0.969^*^	−0.045 (0.014)	0.956^**^	−0.027 (0.014)	0.974^*^
Stoic endurance	−0.161 (0.114)	0.852	−0.084 (0.133)	0.920	−0.073 (0.117)	0.930
Stoic taciturnity	0.028 (0.134)	1.029	−0.019 (0.135)	0.981	0.063 (0.117)	1.065

*Note*:

* *P* ≤ 0.05,

** *P* ≤ 0.01.

**Table 5.** Results of fully adjusted model, feeling subjectively worse

Feeling subjectively worse						
	Friends/family		Strangers		Gregarious	
	Coef. (SE)	Exp (coef.)	Coef. (SE)	Exp (coef.)	Coef. (SE)	Exp (coef.)
Age	−0.019 (0.011)	0.981	−0.026 (0.011)	0.974^*^	−0.019 (0.009)	0.981^*^
Female	−0.497 (0.261)	0.608	0.091 (0.285)	1.096	−0.652 (0.238)	0.521^**^
Non-Hispanic Black	−0.255 (0.207)	0.775	−0.282 (0.234)	0.754	−0.684 (0.208)	0.505^**^
Hispanic	0.024 (0.238)	1.024	−0.181 (0.223)	0.834	−0.483 (0.213)	0.617^*^
Currently married	0.425 (0.231)	1.530	0.159 (0.217)	1.173	0.291 (0.186)	1.338
SicknessQ	0.027 (0.012)	1.027^*^	0.006 (0.015)	1.006	0.032 (0.011)	1.032^**^
Sick attention	0.214 (0.083)	1.239^**^	0.167 (0.094)	1.181	0.259 (0.085)	1.295^**^
Gender role	0.065 (0.065)	1.067	−0.007 (0.069)	0.993	0.131 (0.060)	1.140^*^
Machismo	0.187 (0.085)	1.206^*^	0.175 (0.084)	1.191^*^	0.316 (0.076)	1.371^**^
Familism	−0.148 (0.098)	0.863	−0.060 (0.138)	0.942	−0.144 (0.138)	0.866
Individualism	−0.148 (0.090)	0.862	0.048 (0.076)	1.049	0.042 (0.068)	1.043
Collectivism	0.112 (0.097)	1.118	−0.067 (0.083)	0.936	−0.025 (0.069)	0.975
John Henryism	−0.010 (0.013)	0.990	0.004 (0.015)	1.004	−0.005 (0.013)	0.995
Stoic endurance	0.077 (0.113)	1.081	0.083 (0.122)	1.087	0.060 (0.112)	1.062
Stoic taciturnity	0.122 (0.118)	1.130	0.085 (0.137)	1.089	−0.004 (0.122)	0.996

*Note*:

* *P* ≤ 0.05,

** *P* ≤ 0.01.

**Figure 2.**
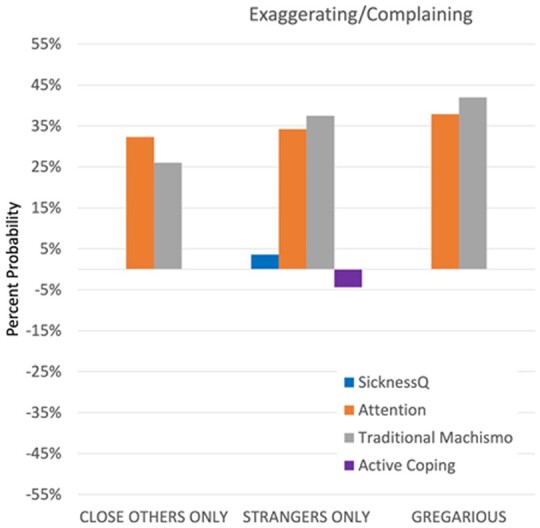
Significant predictors of communication style, exaggerating/complaining about symptoms

Age was also associated with a reduced probability of exaggerating or complaining about sickness to friends and family as well as a gregarious style (Table 4). Additional demographic factors associated with reduced gregariousness relative to taciturnity were female sex (exp.(b) = 0.405, 59% decrease) and non-Hispanic Black race/ethnicity (exp.(b) = 0.537, 46.3% decrease); Table 4). Marital status was associated with an increased probability of complaining/exaggerating to strangers only. Similar to the previous signaling domain, greater sickness attention scores were uniformly associated with greater complaining/exaggerating (Table 4, Fig. 2). Contrary to predictions, greater recalled SicknessQ scores were associated with a small increase in the probability of exaggerating/complaining to strangers only (exp.(b) = 1.036, 3.6% increase). Traditional machismo was linked with greater exaggerating/complaining across all outcomes, with the strongest effect for gregariousness (exp.(b) = 1.420, 42% increase). Finally, an active coping style (i.e. John Henryism) was associated with a small decrease in the probability of exaggerating/complaining to strangers (exp.(b) = 0.956, 4.4% decrease).

**Figure 3.**
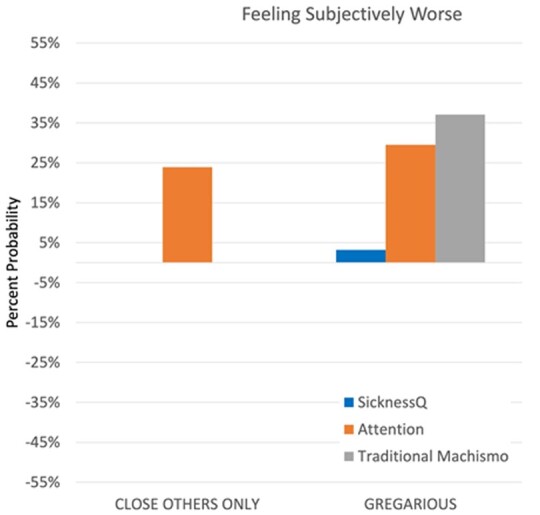
Significant predictors of communication style, feeling subjectively worse

Table 5 shows the results for subjective sensations of sickness. Female sex and non-Hispanic Black race/ethnicity were both linked with reduced gregariousness in this domain. A greater sickness attention score was again associated with an increased probability of signaling to friends and family only and a gregarious style. Greater recalled SicknessQ scores were associated with worse subjective sensations when among both friends/family and strangers (exp.(b) = 1.032, 3.2% increase) at the 0.01 significance level but also among friends/family only at the 0.05 level. Notably, the significant relationship between SicknessQ and feeling subjectively worse around friends/family found in the baseline model was not found after adding the cultural variables to the model, suggesting that this effect is explained by one of the latter factors. Traditional machismo was linked with a greater probability of gregariousness (Table 5, Fig. 3).

## DISCUSSION

In a large, nationwide survey, we found that many respondents were more likely to talk about their symptoms, exaggerate or complain about their symptoms, or feel subjectively worse with their friends and family rather than strangers although the majority were reportedly taciturn and preferred to keep their sickness to themselves; that attention received from others while sick was generally associated with an increased probability of sickness signaling even after adjusting for cultural factors; and that self-reported, recalled sickness behavior was associated with some communication styles, though cultural factors appear to explain at least one of these relationships. Taken together, these results lend some support to a signaling function of sickness behavior, though we cannot entirely rule out other explanations. The degree to which our categories of sickness communication overlap with existing communication styles is not known. Additional studies that control for overall communication style will be able to disentangle this. Nevertheless, the connections between sickness communication style and sickness behavior found in our analyses are suggestive.

We predicted that sickness behavior would be associated with a greater probability of communicating sickness to close others (i.e. friends and family). Consistent with this, we found that self-reported, recalled sickness behavior was positively associated with feeling subjectively worse around close others, though this effect disappeared when adding sociocultural variables to our model. Contrary to predictions, we found stable relationships between sickness behavior and signaling to strangers or adopting a gregarious communication style. Specifically, sickness behavior was associated with exaggerating/complaining to strangers only and with worse subjective sensations when around others, whether family and friends or strangers. One possible explanation for sickness behavior’s association with such gregariousness is that the time and energy spent on social interactions when sick simply leads to feeling worse, a reversal of our proposed causality.

On the other hand, the largely consistent associations between signaling and attention received suggest that there is a positive benefit to these behaviors for the sick person. It seems likely that received attention reinforces the sickness signaling strategy; signals that do not result in a net benefit for the signaler are not likely to be maintained or selected for. Note that this measure of attention is only a single item and does not specify what type of attention is received, whether it be caregiving, simple expressions of concern and so on.

We also found a role for sociocultural norms/values in sickness signaling. When adding these variables to our models, the relationship between SicknessQ scores and feeling subjectively worse around close others disappeared, suggesting that this association is entirely explained by sociocultural factors. One dimension of stoicism, enduring pain and illness, was associated with a reduced likelihood of talking about sickness with others, particularly friends and family, a result in line with other research on stoicism and help-seeking [26]. Additionally, an active coping style was associated with a reduced likelihood of exaggerating or complaining around strangers. This is unsurprising, given that active coping, as measured in this study with the John Henryism scale, focuses on the individual’s ability to deal with or otherwise control their problems on their own [38].

Contrary to our tentative predictions, we found that traditional machismo was positively associated with signaling in multiple domains, particularly in exaggerating sickness. Like stoicism, machismo is sometimes associated with reduced preventative health behaviors, including screening exams [43]. However, an increased propensity to talk about, exaggerate or complain about symptoms may reflect a desire to project a powerful and/or prestigious self-image by indicating that one is able to overcome sickness. Given the relative importance of dominance/power and prestige in the lives of men in America and elsewhere, the benefits of projecting this self-image are apparent [44]. However, women in our sample also completed the traditional machismo instrument. Health and illness may also be related to prestige or power in Western women. In a content analysis of health articles in Canadian women’s magazines, Roy [45] notes that messaging aimed at women often concerns personal responsibility and overcoming illness, and links good health to moral worth as both a citizen and a woman. It is possible that talking about and exaggerating symptoms may play into this narrative of overcoming sickness for the women in our sample. Finally, with regard to the connection between traditional machismo and experiencing worse subjective symptoms in gregarious contexts, it may be that talking about or otherwise dwelling on somatic symptoms increases interoceptive awareness (i.e. the perceptual accuracy hypothesis) and hence symptom severity ratings [46].

Individualism was associated with talking about sickness to friends/family and gregariously. Those high in individualism tend to have more universal or abstract prosocial values, which may translate into helping in-group and out-group members equally, such as notifying someone that they feel sick and perhaps avoiding shaking hands or other close contact [47]. Familism was negatively associated with talking about sickness with strangers only, perhaps reflecting a preference for sharing health information with family members only. There were several relations between sociocultural factors and sickness signaling across analyses at the 0.05 level, but these were no longer related when we applied a more stringent *P-*value criterion to minimize the risk of Type 1 errors in our models. For instance, active coping and traditional machismo were also associated with additional aspects of signaling at the 0.05 significance level, consistent with the above descriptions.

Certain demographic factors were also associated with communication style. Increased age was associated with less likelihood of talking about sickness or exaggerating/complaining. This may reflect greater self-sufficiency at older ages or perhaps some degree of generational differences in norms surrounding discussing or otherwise communicating sickness. Women were less likely to report increased subjective symptom severity or exaggerate their symptoms around all others. This is in agreement with experimental results showing that men tend to overexaggerate common cold symptoms and that men have a lower threshold for expressing discomfort following an LPS injection, suggesting that men communicate about sickness more readily than women [5]. Interestingly, a stronger feminine gender role, independent of identifying as a man or a woman, was associated with an increased likelihood of feeling subjectively worse and exaggerating symptoms around all others at the 0.05 significance level. The disconnection between gender identification and culturally bound gender roles as they relate to health outcomes warrants further attention [29, 48]. Ballering et al. [45] found that a greater feminine gender role was associated with increased self-reported somatic symptoms, particularly in men. Similarly, Annandale and Hunt [29] reported that both men and women who scored as more feminine on the Bem Sex Role Inventory reported more mental and physical symptoms, Finally, we found that non-Hispanic Blacks were less likely to exaggerate symptoms or feel subjectively worse around others, relative to non-Hispanic whites. This relative taciturnity might reflect previously described patterns of medical mistrust and/or an unwillingness to look weak that shape physical and mental illness experiences [49,50]. Future research is needed to understand how demographics and cultural factors interact to shape sickness communication.

## CONCLUSIONS AND IMPLICATIONS

In sum, our analyses found some tentative support for a signaling function for sickness behavior, although results contradicted to our predictions of greater signaling to close others only. Signaling behavior is perhaps reinforced by successfully receiving attention in the past. We also find evidence that some demographic and sociocultural factors affect signaling styles and moderate at least one relationship between sickness behavior and signaling. There are several limitations to the study, including its self-reported nature and the use of a recalled measure of sickness behavior. Further, while many compelling effects emerged, many effect sizes were modest, particularly for relationships with SicknessQ. Asking more detailed questions about the exact type of attention or assistance given to individuals when they are sick would be helpful. Experimental studies will be able to manipulate social interactions to better test predictions about signaling and will be able to collect data when participants are actively experiencing sickness behavior, rather than relying on recall. Additionally, experimental studies that vary the dose of LPS received can determine whether the nature of a sickness signal varies based on the severity of the illness [14]. Theoretically, such signals should be easier to detect and carry more valence for the recipient if the severity of the illness and the risk of transmission are high [14].

To our knowledge, this is one of the first attempts to explicitly connect sickness behavior and signaling in humans. Given the fundamental nature of energy use by the immune system, it seems likely that any signaling function of sickness behavior is an exaptation . This does not mean that such a signaling function is inconsequential. Indeed, communicating sickness—and acting upon that signal—have been a central social interaction throughout the history of our species and contributed to our substantial success . These signals are no less important today, though their meaning may be bolstered or obscured through complex cultural lenses.

## Supplementary data

Supplementary data is available at *EMPH* online.

## Supplementary Material

eoab017_Supplementary_Data
