# Supplementary material for: Signaling sickness: the role of recalled sickness behavior and psychosocial factors in shaping communication style
Source: Evol Med Public Health. 2021 Jun 11;9(1):221–31. doi: 10.1093/emph/eoab017 (PMC8364984; doi:10.1093/emph/eoab017)
Supplement: eoab017_Supplementary_Data [file eoab017_supplementary_data.docx]

**SUPPLEMENTARY MATERIALS**

**Results of Baseline Multinomial Probit Model, Talking About Symptoms**

| **Talking about Sickness** | | | | | | |
| --- | --- | --- | --- | --- | --- | --- |
|  | **Friends/Family** | | **Strangers** | | **Gregarious** | |
|  | Coef. (SE) | Exp(coef.) | Coef. (SE) | Exp(coef.) | Coef. (SE) | Exp(coef.) |
| Age | -0.014  (0.009) | 0.986 | -0.039 (0.014) | 0.962^**^ | -0.090  (0.010) | 0.991 |
| Female | 0.176  (0.167) | 1.192 | -0.492  (0.209) | 0.611^*^ | -0.879  (0.176) | 0.415^**^ |
| Non-Hispanic Black | 0.080  (0.187) | 1.083 | -0.016  (0.275) | 0.984 | -0.033  (0.232) | 0.968 |
| Hispanic | 0.066  (0.195) | 1.069 | -0.374  (0.348) | 0.688 | 0.001  (0.238) | 1.001 |
| Currently Married | 0.210  (0.182) | 1.234 | 0.623  (0.263) | 1.864^*^ | 0.793  (0.200) | 2.210^**^ |
| SicknessQ | 0.004  (0.010) | 1.004 | 0.018  (0.015) | 1.018 | 0.028  (0.011) | 1.028^*^ |
| Sick Attention | 0.355  (0.065) | 1.427^**^ | 0.174  (0.087) | 1.190^*^ | 0.571  (0.079) | 1.770^**^ |
| Note: ^*^p ≤ 0.05, ^**^p ≤ 0.01 | | | | | | |

**Results of Baseline Multinomial Probit Model, Exaggerating/Complaining About Symptoms**

| **Exaggerating Sickness** | | | | | | |
| --- | --- | --- | --- | --- | --- | --- |
|  | **Friends/Family** | | **Strangers** | | **Gregarious** | |
|  | Coef. (SE) | Exp(coef.) | Coef. (SE) | Exp(coef.) | Coef. (SE) | Exp(coef.) |
| Age | -0.029  (0.010) | 0.972^**^ | -0.017  (0.011) | 0.983 | -0.024  (0.010) | 0.976^*^ |
| Female | -0.203  (0.187) | 0.816 | -0.668  (0.212) | 0.513^**^ | -1.073  (0.180) | 0.342^**^ |
| Non-Hispanic Black | 0.133  (0.226) | 1.142 | 0.350  (0.240) | 1.419 | -0.471  (0.223) | 0.625^*^ |
| Hispanic | -0.019  (0.238) | 0.981 | -0.371  (0.251) | 0.690 | -0.440  (0.238) | 0.644 |
| Currently Married | 0.575  (0.129) | 1.778^**^ | 0.830  (0.232) | 2.294^**^ | 0.857  (0.196) | 2.356^**^ |
| SicknessQ | 0.008  (0.115) | 1.008 | 0.031  (0.012) | 1.031^**^ | 0.017  (0.011) | 1.018 |
| Sick Attention | 0.301  (0.072) | 1.352^**^ | 0.323  (0.076) | 1.382^**^ | 0.483  (0.081) | 1.621^**^ |
| Note: ^*^p ≤ 0.05, ^**^p ≤ 0.01 | | | | | | |

**Results of Baseline Multinomial Probit Model, Feeling Subjectively Worse**

| **Feeling Subjectively Worse** | | | | | | |
| --- | --- | --- | --- | --- | --- | --- |
|  | **Friends/Family** | | **Strangers** | | **Gregarious** | |
|  | Coef. (SE) | Exp(coef.) | Coef. (SE) | Exp(coef.) | Coef. (SE) | Exp(coef.) |
| Age | -0.017  (0.011) | 0.983 | -0.024  (0.011) | 0.976^*^ | -0.017  (0.009) | 0.983 |
| Female | -0.488  (0.191) | 0.614^*^ | -0.150  (0.216) | 0.861 | -0.758  (0.171) | 0.469^**^ |
| Non-Hispanic Black | -0.251  (0.209) | 0.778 | -0.238  (0.232) | 0.789 | -0.586  (0.197) | 0.557^**^ |
| Hispanic | -0.147  (0.236) | 0.863 | -0.217  (0.228) | 0.805 | -0.613  (0.210) | 0.542^**^ |
| Currently Married | 0.530  (0.226) | 1.698^*^ | 0.220  (0.228) | 1.246 | 0.513  (0.187) | 1.670^**^ |
| SicknessQ | 0.033  (0.012) | 1.034^**^ | 0.011  (0.014) | 1.011 | 0.040  (0.010) | 1.040^**^ |
| Sick Attention | 0.194  (0.068) | 1.214^**^ | 0.182  (0.085) | 1.199^*^ | 0.341  (0.076) | 1.406^**^ |
| Note: ^*^p ≤ 0.05, ^**^p ≤ 0.01 | | | | | | |
